# Natural selection plays a significant role in governing the codon usage bias in the novel SARS-CoV-2 variants of concern (VOC)

**DOI:** 10.7717/peerj.13562

**Published:** 2022-06-23

**Authors:** Neetu Tyagi, Rahila Sardar, Dinesh Gupta

**Affiliations:** 1 Translational Bioinformatics Group, International Centre for Genetic Engineering and Biotechnology (ICGEB), New Delhi, India, New Delhi, New Delhi, India; 2 Regional Centre for Biotechnology, Faridabad, Haryana, India; 3 Biochemistry, Jamia Hamdard University, New Delhi, New Delhi, India

**Keywords:** Codon usage bias, Mutational pressure, Natural selection, Variants of concern (VOC), SARS-CoV-2

## Abstract

The ongoing prevailing COVID-19 pandemic caused by SARS-CoV-2 is becoming one of the major global health concerns worldwide. The SARS-CoV-2 genome encodes spike (S) glycoprotein that plays a very crucial role in viral entry into the host cell *via* binding of its receptor binding domain (RBD) to the host angiotensin converting enzyme 2 (ACE2) receptor. The continuously evolving SARS-CoV-2 genome results in more severe and transmissible variants characterized by the emergence of novel mutations called ‘variants of concern’ (VOC). The currently designated alpha, beta, gamma, delta and omicron VOC are the focus of this study due to their high transmissibility, increased virulence, and concerns for decreased effectiveness of the available vaccines. In VOC, the spike (S) gene and other non-structural protein mutations may affect the efficacies of the approved COVID-19 vaccines. To understand the diversity of SARS-CoV-2, several studies have been performed on a limited number of sequences. However, only a few studies have focused on codon usage bias (CUBs) pattern analysis of all the VOC strains. Therefore, to evaluate the evolutionary divergence of all VOC S-genes, we performed CUBs analysis on 300,354 sequences to understand the evolutionary relationship with its adaptation in different hosts, *i.e*., humans, bats, and pangolins. Base composition and RSCU analysis revealed the presence of 20 preferred AU-ended and 10 under-preferred GC-ended codons. In addition, CpG was found to be depleted, which may be attributable to the adaptive response by viruses to escape from the host defense process. Moreover, the ENC values revealed a higher bias in codon usage in the VOC S-gene. Further, the neutrality plot analysis demonstrated that S-genes analyzed in this study are under 83.93% influence of natural selection, suggesting its pivotal role in shaping the CUBs. The CUBs pattern of S-genes was found to be very similar among all the VOC strains. Interestingly, we observed that VOC strains followed a trend of antagonistic codon usage with respect to the human host. The identified CUBs divergence would help to understand the virus evolution and its host adaptation, thus help design novel vaccine strategies against the emerging VOC strains. To the best of our knowledge, this is the first report for identifying the evolution of CUBs pattern in all the currently identified VOC.

## Introduction

The continuously mutating SARS-CoV-2 poses significant harm to public health and has emerged worldwide. The World Health Organization (WHO) has classified the variants as variant of concern (VOC), based on a few characteristics such as increased transmissibility, increased virulence, and decreased effectiveness of the available vaccines (WHO, 2021). Therefore, sustained monitoring and rapid assessment of the emerging variants are necessary for the healthcare management of COVID-19.

**Figure 1:**
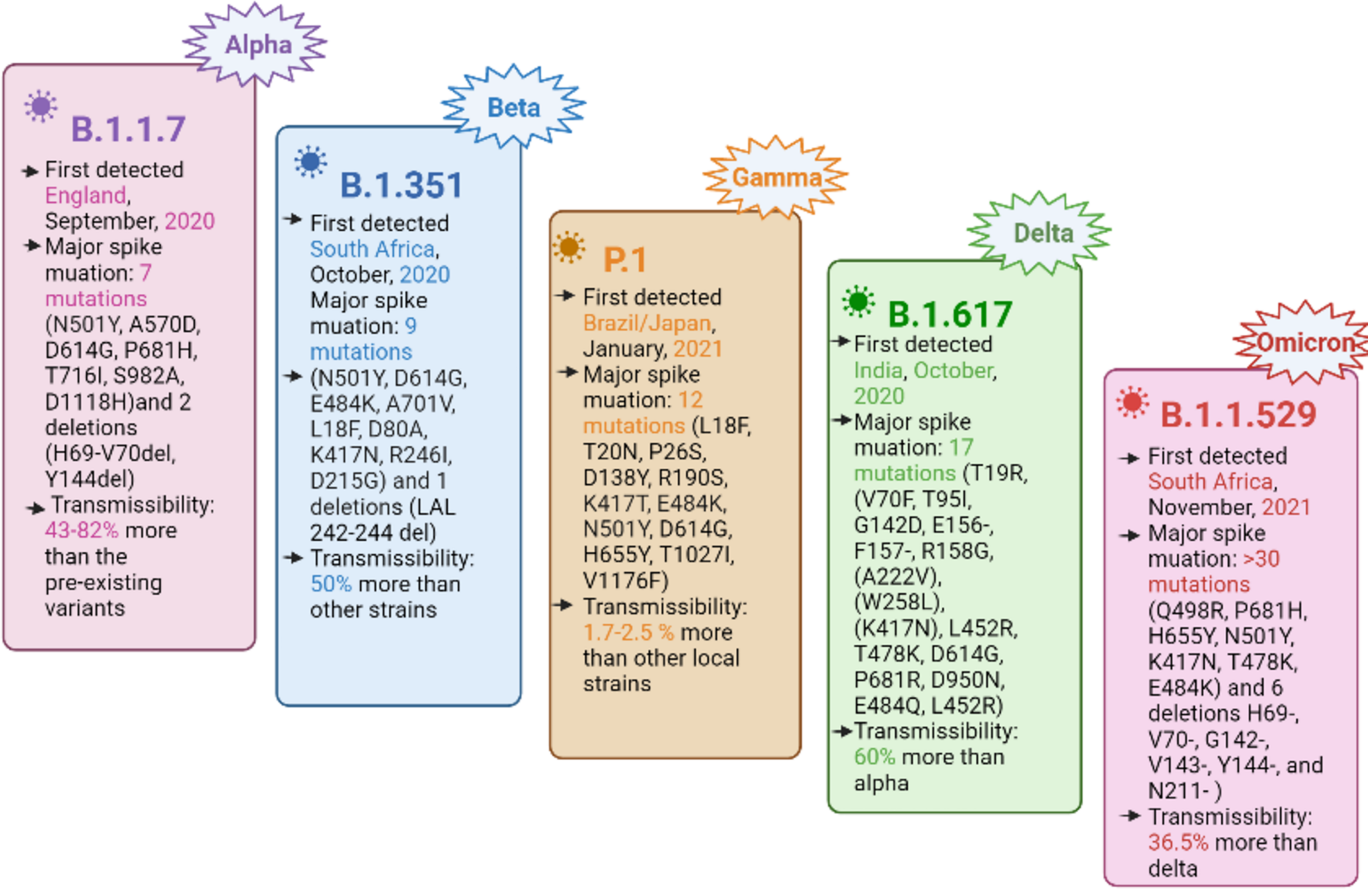
Important characteristics of currently designated SARS-CoV-2 VOC, with spike protein mutations and their transmissibility.

The SARS-CoV-2 genome consists of four structural genes, namely, nucleocapsid phosphoprotein (N) or ribonucleoprotein, a membrane protein (M), the envelope protein (E), and a spike (S) glycoprotein (Malik et al., 2021). The spike glycoprotein facilitates the virus entry into the host cell by binding its receptor binding domain (RBD) to the human cell surface receptor, *i.e*., angiotensin converting enzyme 2 (ACE2) and subsequently, the membrane fusion takes place (Hulswit, de Haan & Bosch, 2016; Yan et al., 2020; Li et al., 2003; Li et al., 2005). Currently, the mutations in the spike protein are of primary concern, leading to harmful consequences in viral pathogenesis by immune invasion and the ineffectiveness of the developed vaccines (Rambaut et al., 2020; Tegally et al., 2021; Faria et al., 2021; Naveca et al., 2021). SARS-CoV-2 is evolving with the continuous accumulation of mutations during replication, resulting in different variants, including VOC. The VOC cause an increase in virus transmissibility, virulence, disease severity, and a potential to reduce the effectiveness of the currently available COVID-19 vaccine, thus becoming a global challenge for the COVID-19 diagnostics and clinical management (Government of Canada, 2022; Berry et al., 2020). The currently designated VOC include alpha (B.1.1.7), beta (B.1.351), gamma (P.1), delta (B.1.617.2), and the recently emerging omicron (B.1.1.529) (Tegally et al., 2021; Supasa et al., 2021; GISAID, 2022; Boehm et al., 2020; Dejnirattisai et al., 2021; Srivastava et al., 2021). A summary of the important VOC characteristics is shown in Fig. 1.

The codon degeneracy leads to the use of different codons for the same amino acid for a particular gene. The preference for one codon over the other is found in different organisms that ultimately lead to the bias of one codon over the other, known as codon usage bias (CUBs) (Komar, 2016). Further, the shape of the codon usage bias is governed by various evolutionary constraints, including mutational pressure, selection pressure, nucleotide composition, dinucleotide frequency and GC content, *etc*. CUBs are determinants of natural selection and important factors controlling gene expression.

To identify the factors shaping CUBs, we performed a comprehensive CUBs analysis on 300,354 SARS-CoV-2 VOC S-genes. Various CUBs properties were calculated, including base composition, GC3 content at each position of the codon, the effective number of codon (ENC), relative synonymous codon usage (RSCU) values, codon adaptation index, dinucleotide frequency, *etc*. were calculated. To the best of our knowledge, this is the first large scale study to analyze CUBs properties for VOC S-genes.

## Materials and Methods

### Retrieval of genomic sequences

The complete high coverage nucleotide sequences of SARS-CoV-2 VOC and the other sequence data were retrieved from the National Centre for Biotechnology Information (NCBI Virus portal, https://www.ncbi.nlm.nih.gov/labs/virus/vssi/#/sars-cov-2). Further, we extracted the S-gene sequences from the complete genomes, available till October 21st, 2021. For omicron, the complete nucleotide sequences were retrieved on April 5th, 2022. S gene reference sequences for SARS-CoV-2 (NC_045512.2), SARS-CoV (NC_004718.3), MERS-CoV (NC_01984.3) and complete genome of host *H. sapiens* (GRCh38.p13) were retrieved from the NCBI. Additionally, we retrieved genome sequences for four closely related Bat-CoVs with accession IDs (MG772933, MG772934, MW251308, MN996532) six Pangolin-CoVs with accession IDs MT040333, MT040334, MT040335, MT040336, MT072864, MT121216 from the NCBI virus portal. The list of all the accession numbers of all VOC S-gene sequences are provided in the supplementary Dataset S1.

### Nucleotide composition

The nucleotide composition for all the VOC S-gene sequences was calculated. The computed properties include the frequency of each nucleotide at the third position of the synonymous codons (A3s, T3s, G3s, C3s); overall A, T, G, C (base composition) and G+C content at 1^st^, 2^nd^ and 3^rd^ codon positions GC1, GC2, GC3, respectively. The complete nucleotide composition for VOC, bat-CoVs and pangolin-CoVs details can be obtained from Table S1.

### RSCU analysis and heatmap generation

To investigate the factors affecting the synonymous codon usage bias, RSCU values were calculated using CodonW (http://codonw.sourceforge.net/). The RSCU values were calculated using the formula:

${\chi}={X_{ij}\over \sum\nolimits_{j}^{n_{i}}X_{ij}}n_i$

*X_ij_* represents the number of *i*th codons for the *j*th amino acid, and *n_i_* represents the degenerate number of a specific synonymous codon, ranging from 1 to 61.

High RSCU is the ratio of observed to the expected value for given amino acid and its value is not affected by the length of the sequence or amino acid frequency (Sharp & Li, 1986). A higher RSCU value (RSCU > 1) indicates positive codon bias and is considered a preferred codon, whereas the lower RSCU value (RSCU < 1) represents the negative codon bias termed as under-preferred codons. The RSCU values for all VOC S-gene sequences, SARS-CoV-2, SARS-CoV, MERS-CoV, bat-CoVs, pangolin-CoVs and the host (*H. sapiens*) were compared and visualized with a heatmap in R. The stop codons (UGA, UAG, UAA) and amino acids bearing single codons (AUG, and UGG) were excluded in this study.

### Dinucleotide frequency analysis

The dinucleotide frequency calculation in a genome can be used to estimate CUBs. We have calculated the dinucleotide frequency for all the VOC S-gene sequences. The average relative abundance value for each dinucleotide was determined by the odds ratio, defined as the ratio of observed and expected dinucleotide frequencies. The odds ratio value >1.23 was considered over-represented, whereas the value <0.78 as under-represented (Zhou & Chen, 2012).

### ENC analysis

The ENC-plot was generated by plotting the ENC values against the GC3 values to further investigate the synonymous codon usage pattern. The ENC is used to measure the deviation from the random codon usage pattern; its value ranges from 20–61. A lower ENC value (<35) corresponds to strong codon usage bias, whereas higher ENC values (>35) represent low codon bias (Wright & Fortran, 1990). The standard ENC values were calculated using the formula,

${ENC_{expected}}=2+S+{29\over{(s^2+{(1-s)^2})}}$

*S* represents the given GC3s value. If the genes lie on or just below the standard curve, the CUB is determined by mutational pressure. Alternatively, if a particular gene is subjected to natural selection, it falls below the standard curve. The relative extent of natural selection or mutational pressure affecting the CUBs can be measured by the distance between the point where the gene lies and the standard curve.

### Neutrality plot analysis

Codon usage disparity is governed mainly by two important factors, mutation pressure and natural selection. In neutrality plot analysis, the main factors affecting the CUBs were determined by taking the mean GC content at the 1^st^ and 2^nd^ position (GC12 x-axis) and plotted against GC content at the 3^rd^ position of the codon (GC3 y-axis) values. Plotting GC12 values against GC3s helps analyze the correlation between the base compositions of all three codon sites, thus determining the main factor responsible for the codon usage bias. The regression line’s slope indicates the effect of mutational pressure (Sueoka, 1988). The correlation between GC12 and GC3 measures the relative extent to which natural selection or mutational pressure affects the CUBs of the particular gene.

### Software and tools used

CodonW was used to calculate various CUBs related properties, such as RSCU values, ENC calculation, and other codon usage indices (http://codonw.sourceforge.net/). EMBOSS program was used to calculate the mean GC content at the 1^st^ and 2^nd^ position (GC12) and GC content at the third position of codon (GC3) using EMBOSS cusp. For dinucleotide frequency and Codon Adaptation Index (CAI) calculation, EMBOSS Compseq and EMBOSS CAI program was used (http://emboss.sourceforge.net/).

## Results

In total, 300,354 complete nucleotide sequences from the NCBI virus portal were collected, including 102,298, 6,727, 3,050, 447, 79,143, and 8,678 for the alpha, gamma, delta, beta, and omicron BA.1 and BA.2 lineages, respectively. Further, the S-gene sequences were extracted to calculate different codon usage indices.

### Nucleotide composition and codon usage indices of VOC

The nucleotide composition was calculated for all the VOC S-gene sequences analyzed here. The nucleotide composition was found to be in the order of U > A > C > G (see Table S1). The nucleotides at the 3^rd^ position of the codon also follow a similar trend U_3_ > A_3_ > C_3_ > G_3_ (see Table 1). The average GC content was 0.37, with a standard deviation of 0.0007. The mean CAI was 0.646, with a standard deviation of 0.0009.

**Table 1:** The codon usage indices of S genes in VOC.

	U3s	C3s	A3s	G3s	ENC	GC3s	GC	GC12	CAI
Alpha	0.548	0.189	0.377	0.134	44.298	0.267	0.373	42.555	0.646
Beta	0.548	0.189	0.377	0.132	44.181	0.265	0.373	42.375	0.646
Gamma	0.549	0.188	0.378	0.132	44.105	0.266	0.371	42.355	0.646
Delta	0.549	0.188	0.378	0.132	44.341	0.265	0.373	42.605	0.645
Omicron_BA.1	0.547	0.187	0.381	0.136	44.738	26.560	0.372	42.215	0.648
Omicron_BA.2	0.549	0.187	0.379	0.136	44.669	26.630	0.372	42.370	0.647
SARS-CoV-2	0.548	0.189	0.377	0.133	44.160	0.267	0.373	42.620	0.646
MERS	0.547	0.227	0.278	0.183	47.690	35.250	0.409	43.795	0.660
SARS-CoV	0.548	0.214	0.332	0.149	45.730	30.100	0.388	43.115	0.664
Host	0.374	0.271	0.365	0.245	52.458	38.870	0.427	38.875	0.688

### Codon usage pattern of VOC S-genes

**Figure 2:**
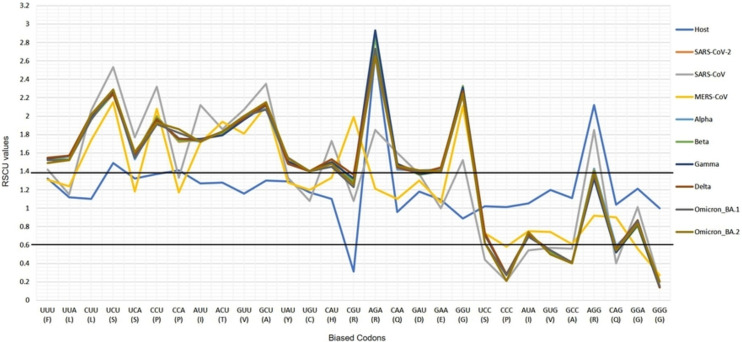
The profiles of the RSCU values of SARS-CoV-2 VOC S genes, the over and under-preferred codons (RSCU difference ≥0.20 between the host *H. sapiens* and VOC), the bold horizontal lines showing the categorization of RSCU values >1.4 (highly preferred codons), and the RSCU values <0.6 (highly under-preferred codons).

**Figure 3:**
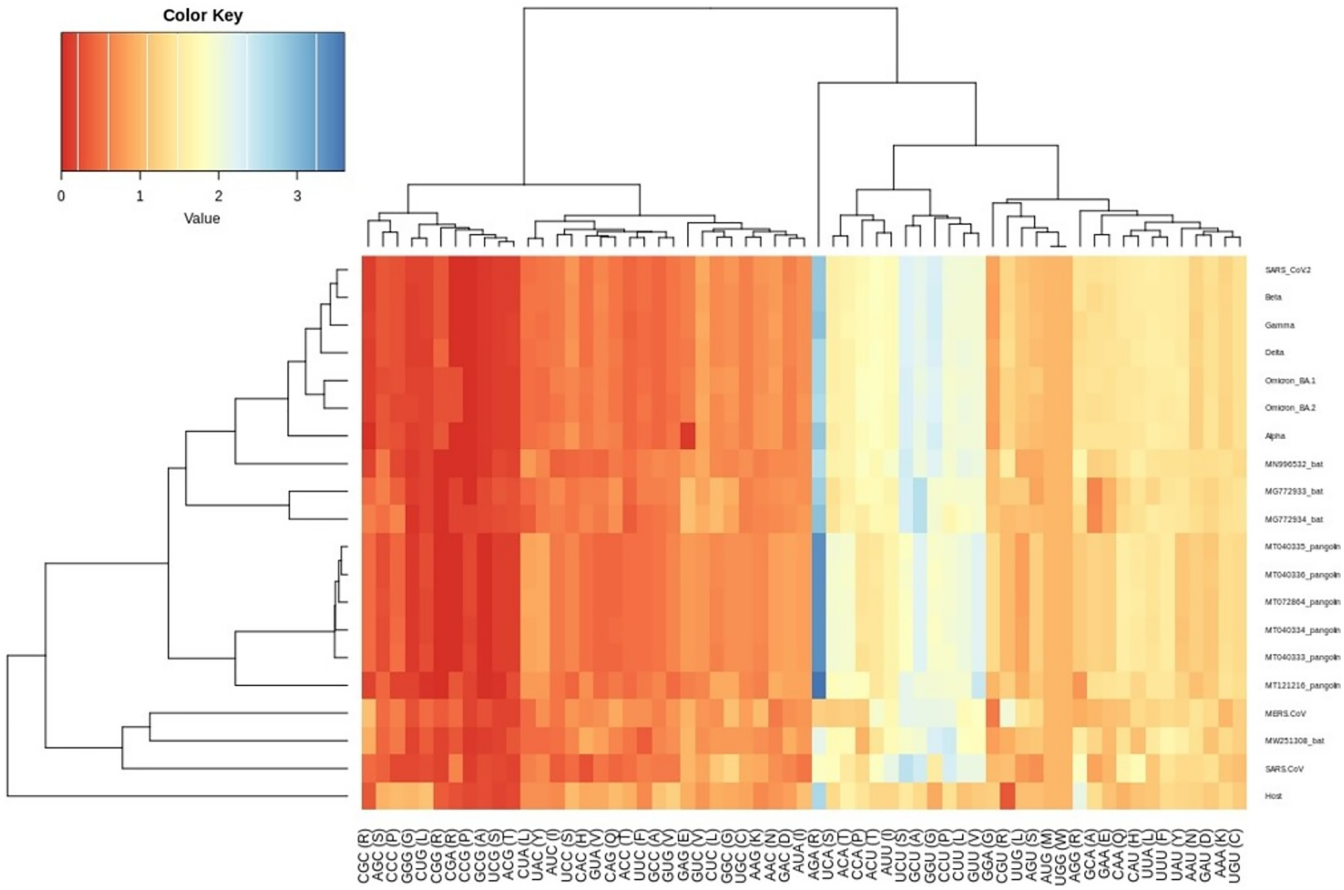
Heatmap of RSCU values for SARS-CoV-2 VOC S genes with respect to the host. Each column represents a codon. The colour key RYB: red, yellow, blue represent the lower to higher RSCU values, respectively.

The CUBs exists in many RNA viral genomes, generally determined by mutation and selection pressure. RSCU analysis was performed to investigate the codon usage bias pattern in all the VOC S-genes. When compared with *H. sapiens* host, we found 20 preferred codons (UUU (F), UUA (L), CUU (L), UCU (S), UCA (S), CCU (P), CCA (P), AUU (I), ACU (T), GUU (V), GCU (A), UAU (Y), UGU (C), CAU (H), CGU (R), AGA (R), CAA (Q), GAU (D), GAA (E), and GGU (G)) in all the VOC S-genes. Out of the 20 preferred codons, 14 are U-ending, while six are A-ending. Among them, 10 codons, *i.e*., AUU (I), ACU (T), GCU (A), GAA (E), GGU (G), UUA (L), CUU (L), UCU (S), CCU (P), CCA (P) were found to be highly preferred. These highly preferred codons were found to exhibit antagonism with the host (*H. sapiens)* codon usage patterns. Ten codons were found to be under-preferred or rarely used codons (UCC (S), CUG (L), CCC (P), AUA (I), GUG (V), GCC (A), AGG (R), CAG (Q), GGA (G), GGG (G)), comprising of 5-G ending, 3-C ending, and 2-A ending (see Table 2). Many of the identified codons are previously reported as preferred codons in various CoV genomes (Sheikh et al., 2019). It was observed that all the VOC spike genes are highly biased towards A/U-ending codons. In contrast, the under-preferred codons were mostly C/G ending (Dutta, Buragohain & Borah, 2020). The preferred and under-preferred codons RSCU values of all the VOC with their respective host were plotted in a line plot (Fig. 2). The RSCU profiling revealed that ten of the twenty preferred codons exhibit antagonism with the human codon usage (see Table 2). The RSCU ranges from 0 (CCG (P), CGC (R), CGA (R)) to 2.93 for Arginine (AGA (R)). The heatmap and the associated clustering of all the codons are shown in Fig. 3. A slight or negligible difference in RSCU values was observed among all S-gene sequences of VOC. Interestingly, the RSCU analysis spotlighted the usage of CCG (P) and nil usage of CGA (R) codons, reported previously for SARS-CoV-2 genomes except for BA.1 and BA.2 omicron sequences (Hou, 2020; Berkhout, 2022). Strikingly, we observed nil usage of CGC (R) by alpha. When comparing the codon usage of VOC S-genes with the bat-CoVs, intriguingly, we observed nil usage of CCG (P) codon and CGA (R) by MN996532_bat genome. This indicates that MN996532 (bat-RaTG13) S-gene codon usage is highly similar to SARS-CoV-2 S-gene, thus clustered together in the heatmap. Previously, it was reported that SARS-CoV-2 is supposed to originate from bat-RaTG13 and the spike protein of RaTG13 and SARS-CoV-2 S protein are closely related (Ratg et al., 2020; Wrobel et al., 2020). With respect to SARS-CoV-2, we observed nil usage of codon CGG (R) and GCG (A) by pangolin-CoVs and CGG (R) codon for bats-CoV except for the MW251308_bat sequence. When comparing RSCU values of bat and pangolin-CoVs with respect to the human host, 12 codons were found to be over and under-preferred, namely CUU (L), UCU (S), CCU (P), AUU (I), ACU (T), GUU (V), GCU (A) CCC (P), AUA (I), GCC (A), AGA (R), and CAG (Q), concordant to other VOC CUBs pattern.

**Table 2:** Relative synonymous codon usage (RSCU) patterns in S genes of VOC in comparison with its host *Homo sapiens*.

Amino acids	Codon	Host	SARS_CoV-2	SARS-CoV	MERS-CoV	Alpha	Beta	Gamma	Delta	Omicron_lineage_BA.1	Omicron_lineage_BA.2
Phenylalanine	**UUU (F)**	**1.32**	**1.53**	**1.42**	**1.31**	**1.53**	**1.53**	**1.54**	**1.55**	**1.52**	**1.49**
	UUC (F)	0.68	0.47	0.58	0.69	0.47	0.47	0.46	0.45	0.48	0.51
Leucine	**UUA (L)**	**1.12**	**1.56**	**1.15**	**1.24**	**1.56**	**1.53**	**1.57**	**1.57**	**1.52**	**1.52**
	UUG (L)	1.06	1.11	0.79	1.44	1.11	1.14	1.12	1.12	1.12	1.12
	**CUU (L)**	**1.1**	**2**	**2.06**	**1.74**	**2**	**1.97**	**1.98**	**2.02**	**1.96**	**2.02**
	CUC (L)	0.92	0.67	1.15	0.7	0.67	0.68	0.65	0.67	0.73	0.67
	CUA (L)	0.7	0.5	0.61	0.44	0.5	0.51	0.51	0.5	0.5	0.5
	*CUG (L)*	*1.1*	*0.17*	*0.24*	*0.44*	*0.17*	*0.17*	*0.17*	*0.11*	*0.17*	*0.17*
Serine	**UCU (S)**	**1.49**	**2.24**	**2.53**	**2.15**	**2.27**	**2.24**	**2.26**	**2.24**	**2.29**	**2.29**
	*UCC (S)*	*1.02*	*0.73*	*0.44*	*0.73*	*0.73*	*0.72*	*0.71*	*0.73*	*0.62*	*0.62*
	**UCA (S)**	**1.32**	**1.58**	**1.77**	**1.18**	**1.53**	**1.58**	**1.55**	**1.58**	**1.55**	**1.61**
	UCG (S)	0.15	0.12	0.19	0.16	0.12	0.12	0.12	0.12	0.12	0.12
	AGU (S)	1.08	1.03	0.69	1.33	1.04	1.03	1.07	1.03	1.17	1.05
	AGC (S)	0.94	0.3	0.38	0.45	0.31	0.3	0.3	0.3	0.25	0.31
Proline	**CCU (P)**	**1.37**	**2**	**2.32**	**2.08**	**1.96**	**1.99**	**1.96**	**1.97**	**1.91**	**1.93**
	*CCC (P)*	*1.01*	*0.28*	*0.21*	*0.58*	*0.28*	*0.28*	*0.28*	*0.28*	*0.27*	*0.21*
	**CCA (P)**	**1.41**	**1.72**	**1.33**	**1.17**	**1.76**	**1.73**	**1.75**	**1.75**	**1.82**	**1.86**
	CCG (P)	0.21	0	0.14	0.17	0	0	0	0	0	0
Isoleucine	**AUU (I)**	**1.27**	**1.74**	**2.12**	**1.71**	**1.72**	**1.74**	**1.75**	**1.74**	**1.73**	**1.72**
	AUC (I)	0.68	0.55	0.35	0.54	0.58	0.55	0.55	0.55	0.58	0.54
	*AUA (I)*	*1.05*	*0.71*	*0.54*	*0.75*	*0.7*	*0.71*	*0.7*	*0.71*	*0.69*	*0.74*
Methionine	AUG (M)	1	1	1	1	1	1	1	1	1	1
Threonine	**ACU (T)**	**1.28**	**1.81**	**1.86**	**1.94**	**1.83**	**1.82**	**1.79**	**1.83**	**1.83**	**1.83**
	ACC (T)	0.91	0.41	0.48	0.69	0.42	0.41	0.38	0.42	0.43	0.43
	ACA (T)	1.6	1.65	1.49	1.19	1.63	1.65	1.67	1.59	1.62	1.62
	ACG (T)	0.2	0.12	0.16	0.18	0.12	0.12	0.17	0.16	0.13	0.13
Valine	**GUU (V)**	**1.16**	**1.98**	**2.07**	**1.81**	**2**	**1.97**	**1.96**	**1.98**	**2**	**2**
	GUC (V)	0.74	0.87	0.84	0.73	0.83	0.86	0.87	0.86	0.83	0.88
	GUA (V)	0.9	0.62	0.53	0.71	0.62	0.66	0.62	0.62	0.63	0.62
	*GUG (V)*	*1.2*	*0.54*	*0.57*	*0.74*	*0.54*	*0.52*	*0.54*	*0.54*	*0.54*	*0.5*
Alanine	**GCU (A)**	**1.3**	**2.13**	**2.35**	**2.11**	**2.08**	**2.15**	**2.12**	**2.13**	**2.07**	**2.15**
	*GCC (A)*	*1.11*	*0.41*	*0.56*	*0.61*	*0.41*	*0.41*	*0.41*	*0.41*	*0.41*	*0.4*
	GCA (A)	1.36	1.37	0.85	0.99	1.42	1.33	1.37	1.37	1.42	1.35
	GCG (A)	0.23	0.1	0.24	0.28	0.1	0.1	0.1	0.1	0.1	0.1
Tyrosine	**UAU (Y)**	**1.29**	**1.48**	**1.33**	**1.28**	**1.48**	**1.49**	**1.51**	**1.48**	**1.55**	**1.54**
	UAC (Y)	0.71	0.52	0.67	0.72	0.52	0.51	0.49	0.52	0.45	0.46
Cysteine	**UGU (C)**	**1.17**	**1.4**	**1.08**	**1.2**	**1.4**	**1.4**	**1.4**	**1.4**	**1.4**	**1.4**
	UGC (C)	0.83	0.6	0.92	0.8	0.6	0.6	0.6	0.6	0.6	0.6
Tryptophan	UGG (W)	1	1	1	1	1	1	1	1	1	1
Histidine	**CAU (H)**	**1.1**	**1.53**	**1.73**	**1.33**	**1.45**	**1.53**	**1.5**	**1.53**	**1.45**	**1.48**
	CAC (H)	0.9	0.47	0.27	0.67	0.55	0.47	0.5	0.47	0.55	0.52
Arginine	**CGU (R)**	**0.31**	**1.29**	**1.08**	**1.99**	**1.29**	**1.29**	**1.32**	**1.36**	**1.23**	**1.26**
	CGC (R)	0.29	0.14	0.46	1.07	0	0.14	0.15	0.14	0.14	0.14
	CGA (R)	0.27	0	0.62	0.45	0	0	0	0	0.26	0.27
	CGG (R)	0.34	0.29	0.15	0.35	0.29	0.29	0.29	0.41	0.27	0.28
	**AGA (R)**	**2.68**	**2.86**	**1.85**	**1.21**	**2.86**	**2.86**	**2.93**	**2.73**	**2.73**	**2.65**
	*AGG (R)*	*2.12*	*1.43*	*1.85*	*0.92*	*1.43*	*1.41*	*1.32*	*1.36*	*1.37*	*1.4*
Glutamine	**CAA (Q)**	**0.96**	**1.48**	**1.6**	**1.1**	**1.42**	**1.48**	**1.48**	**1.44**	**1.46**	**1.46**
	*CAG (Q)*	*1.04*	*0.52*	*0.4*	*0.9*	*0.59*	*0.52*	*0.52*	*0.56*	*0.54*	*0.54*
Asparagine	AAU (N)	1.25	1.23	1.31	1.44	1.22	1.23	1.2	1.24	1.2	1.21
	AAC (N)	0.75	0.77	0.69	0.56	0.78	0.77	0.8	0.76	0.8	0.79
Lysine	AAA (K)	1.32	1.25	1.13	0.99	1.25	1.28	1.28	1.28	1.28	1.26
	AAG (K)	0.68	0.75	0.87	1.01	0.75	0.72	0.72	0.72	0.72	0.74
Aspartate	**GAU (D)**	**1.18**	**1.39**	**1.37**	**1.3**	**1.41**	**1.36**	**1.37**	**1.38**	**1.39**	**1.41**
	GAC (D)	0.82	0.61	0.63	0.7	0.59	0.64	0.63	0.62	0.61	0.59
Glutamate	**GAA (E)**	**1.09**	**1.42**	**1**	**1.06**	**1.42**	**1.4**	**1.4**	**1.44**	**1.39**	**1.4**
	GAG (E)	0.91	0.58	1	0.94	0.1	0.6	0.6	0.56	0.61	0.6
Glycine	**GGU (G)**	**0.89**	**2.29**	**1.52**	**2.11**	**2.31**	**2.33**	**2.31**	**2.27**	**2.23**	**2.24**
	GGC (G)	0.9	0.73	1.27	1.06	0.72	0.72	0.72	0.72	0.76	0.73
	*GGA (G)*	*1.21*	*0.83*	*1.01*	*0.56*	*0.82*	*0.81*	*0.82*	*0.87*	*0.86*	*0.83*
	*GGG (G)*	*1*	*0.15*	*0.2*	*0.27*	*0.14*	*0.14*	*0.14*	*0.14*	*0.15*	*0.2*

**Note:**

Preferred codons (RSCU > 1) with RSCU difference ≥0.2 between host *Homo sapiens* and VOC are shown in bold. For under preferred codons the RSCU difference between host and VOC should be ≥0.2 and the RSCU value should be ≥1 in host; those follow this trend are marked in italics. The highly preferred codons showing antagonism with *H. sapiens* are marked in red.

### Influence of dinucleotide frequency in determining the codon usage bias among VOC S-genes

**Figure 4:**
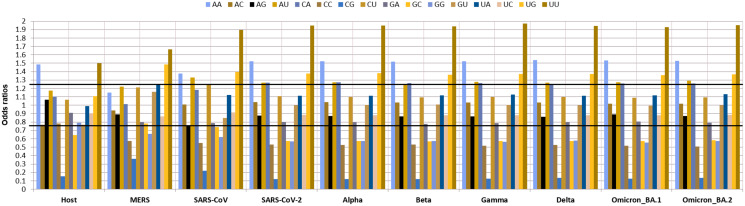
Relative dinucleotide abundance in SARS-CoV-2 VOC S genes in comparison with the host *Homo sapiens*. The bold lines discriminate between over-preferred (odds ratio >1.23) and the under-preferred (odds ratio <0.78).

As dinucleotide usage is another crucial factor in determining the codon usage bias, the relative dinucleotide abundance value for 16 dinucleotide combinations was computed. Interestingly the most abundant dinucleotides across all the VOC were UpU with an odds ratio of 1.94 and ApA with an odds ratio of 1.52 followed by UpG and CpA, with odds ratios of 1.35, 1.37, respectively. Whereas ApU, with an odds ratio of 1.26, was also preferred by the VOC. The GpC (0.57), GpG (0.57), CpC (0.52), and CpG (0.12) were markedly under-represented. Among these CpG (odds ratio: 0.12) was the least abundant dinucleotide observed in all the VOC S-genes. The relative abundance of UpU (1.94) and ApA (1.52) dinucleotides are over-preferred compared to the others (Fig. 4).

### Natural selection, the key driving force of codon usage bias among VOC S-genes

**Figure 5:**
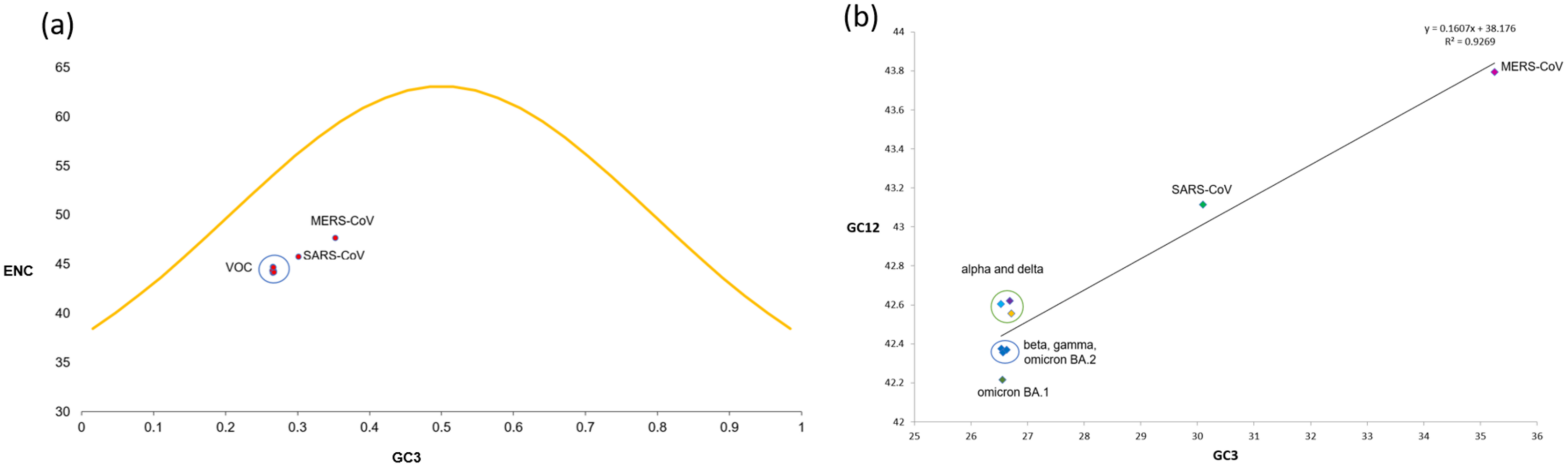
ENC and neutrality plot analysis for SARS-CoV-2 VOC S genes (A) The ENC values are plotted against GC3s; the yellow curve represents the standard curve in the absence of selection. Red dots show different VOC and the host included in the study. (B) Neutrality plot generated by plotting GC content at 1^st^ and 2^nd^ positions of the codon against the GC content at the third position of the codon.

To determine whether mutational pressure or natural selection are the key driving factors affecting the codon bias within the VOC, the ENC-plot was generated by plotting ENC values against the corresponding GC3s values. The ENC-GC3s plot revealed that among all the VOC S-genes, the mean ENC value is 44.35 with a standard deviation of 0.25, suggesting that the codon bias is relatively high across the SARS-CoV-2 VOC S-genes than reported earlier for genome level (Dilucca et al., 2020). Nearly all the dots in ENC-plot were located below the standard curve (Fig. 5A), indicating that the codon bias in all the VOC is majorly due to natural selection and other factors.

### Neutrality plot analysis

The neutrality plot (Fig. 5B) indicates a quite high correlation between the GC12 and GC3 values belonging to the S-genes of different VOC, with r^2^ = 0.926 and significant *p*-value of 0.000106. The regression line’s slope and intercept were calculated to be 0.1607 and 0.8393, respectively, suggesting the contribution of 16.07% and 83.93% by mutational pressure and natural selection. Thus, relative neutrality was calculated to be ~84%.

## Discussion

Codon usage bias analysis provides deep insights into virus evolutionary pressure, host adaptation, and pathogenesis. CUBs pattern has been identified for various viral structural and non-structural genes (Sheikh et al., 2019; Makhija & Kumar, 2015; Alnazawi, Altaher & Kandeel, 2017). Viruses are dependent on host cellular machinery for their replication. CUBs pattern analysis with respect to host has proven valuable in understanding the viral adaptation and evasion of host immune responses (Butt et al., 2016). SARS-CoV-2 strains are significantly similar to pangolin-nCoV, with bat-nCoV being a distant second (Zhao, Cui & Tian, 2020). Several studies have been conducted to understand the diversity of SARS-CoV-2, which were performed on a limited number of samples (Dilucca et al., 2020; Roy et al., 2021; Khattak et al., 2021). The very recent study on the CUBs analysis on highly transmissible delta variant was performed on 159 sequences (Li, Zhang & Xue, 2022).

Furthermore, the previous studies have not focused on the CUBs analysis of VOC S-genes. Therefore, we investigated the factors determining the codon usage divergence in the VOC S-genes in the present study. We performed S-genes CUB analysis on 300,354 genomic sequences with respect to human hosts (*H. sapiens*) and their intermediate hosts, *i.e*., bats and pangolins.

**Figure 6:**
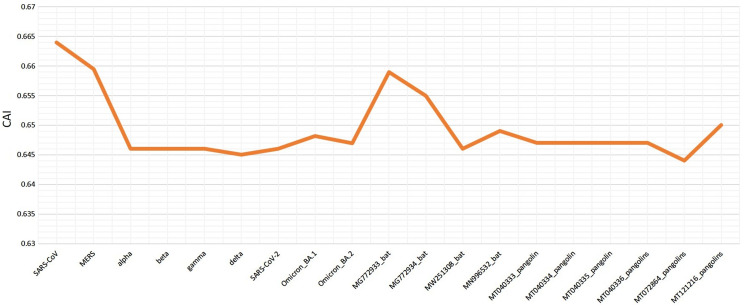
Codon adaptation index (CAI) values for SARS-CoV-2 (S-gene) isolated from different hosts, calculated by taking human as reference.

Various codon usage indices of VOC S-genes were calculated to quantify their adaptability and evolution with respect to different hosts. The nucleotide composition analysis revealed minimal C and G, however a higher A and U nucleotide usage in its genome (Woo et al., 2007; Berkhout & van Hemert, 2015). The higher usage of A and U nucleotides was also found at the 3^rd^ position of the codon. Thus, the VOC S-gene was pyrimidine rich, with >60% of AU nucleotides. The CAI values can be used to measure the extent of adaptability of the virus inside the host, its value ranges from 0 to 1. The higher value reveals higher gene expression and better adaptability in the host (Khodary & Anwar, 2020). When comparing the CAI values of human isolated SARS-CoV-2 VOC with the closely related bat-CoVs and pangolin-CoVs by taking human codon usage as a reference, it was revealed similar pattern of adaptation to the human cellular system of SARS-CoV-2 and the closely related pangolin-CoV (see Fig. 6). However, for the closely related bat-CoVs CAI values were found to be slightly higher.

The RSCU analysis revealed 10 highly preferred codons showing antagonistic behaviour with respect to the human host (see Table 2). A similar pattern was observed in other viruses too, such as malburg virus and hepatitis A virus. Antagonistic codons are involved in the proper folding of the viral proteins in Marburg, hepatitis A and C viruses (Nasrullah et al., 2015; Hu et al., 2011; Bosch & Pinto, 2003).

There were 10 under-preferred codons or rarely used codons (UCC (S), CUG (L), CCC (P), AUA (I), GUG (V), GCC (A), AGG (R), CAG (Q), GGA (G), GGG (G)), comprising of 5-G ending, 3-C ending, and 2-A ending. Analysis of these underrepresented codons can be used to generate live-attenuated vaccines using the synthetic attenuated virus engineering technique. This process involves synthesising a viral genome so that the wild-type amino acid sequence is preserved while existing synonymous codons are rearranged to create a sub-optimal arrangement of codon pairs that are generally under-represented (Roy et al., 2021). Similar approaches have been employed in the development of live attenuated vaccines against poliovirus (Coleman et al., 2009), human respiratory syncytial virus (Le Nouën et al., 2014), influenza virus (Mueller et al., 2011), and dengue virus (Shen et al., 2015). The present study results are similar to that of the previous studies performed on SARS-CoV-2 structural genes, validating our findings (Dilucca et al., 2020; Khodary & Anwar, 2020; Kandeel et al., 2020; Gu et al., 2020; Tort, Castells & Cristina, 2020; Nyayanit et al., 2021).

The RSCU analysis demonstrated that all the VOC use a similar set of codons optimized for their usage with respect to the host. The heatmap dendrogram showed a clear separation between human SARS-CoV-2, bat-CoV and pangolin-CoV S-genes codon usage except for MN996532 (bat-RaTG13), which clustered with VOC. MW251308 shares a clade with MERS-CoV and SARS-CoV (Fig. 3). It has been previously reported that bat-RaTG13 and SARS-CoV-2 share significant similarities (Zhou et al., 2020). With respect to bat-RaTG13, MG772933 and MG772934 are distantly related to SARS-CoV-2. Of the six pangolin-CoV, five grouped together, MT121216 outgrouped from the other pangolins and was observed to be less closer to the SARS-CoV-2 than bat-CoV. In terms of adaptability too, our results showed higher adaptability of SARS-CoV-2 in bats than in pangolins and humans (see Fig. 6).

CUB is influenced by dinucleotide frequency amongst the RNA viruses (Belalov & Lukashev, 2013). The results of our study showed minimal abundance, *i.e*., depletion of dinucleotides CpG. The study by Subramanian revealed that the whole genome of SARS-CoV-2 consists of 1.47% of CpG dinucleotides, which is much lower than the other betacoronaviruses (Subramanian, 2021). Antiviral zinc protein (ZAP) of the host is the main target site suggesting the depletion is due to the adaptive response by viruses to escape from the host defense process (Vetsigian & Goldenfeld, 2009). This reduction in CpG can be helpful in predicting CpG nucleotides in VOC S-genes that constitute epitopes that are possibly mutated to UpG. Hence, identifying these mutations can help design epitopes that recognize different emerging variants of SARS-CoV-2 (Subramanian, 2021). Depletion of CpG dinucleotides has also been associated with virus evolution, replication, adaptation and innate immune responses. Through their interactions with the toll-like receptor-9 (TLR9) and the zinc-finger antiviral protein (ZAP), CpG dinucleotides promote immune responses to inhibit virion formation. For these reasons, they are used as vaccine adjuvants for coronaviruses (Kames et al., 2020).

The frequency of codon usage is not usually random, and it has been linked to translation efficiency, mutational drift, and other selection pressures such as natural selection (Bulmer, 1991; Hershberg & Petrov, 2009; Iriarte, Lamolle & Musto, 2021; Komar, 2016; Musto, 2016; Novoa et al., 2019; Sharp, Emery & Zeng, 2010; Supek, 2016). Among them, mutational pressure and natural selection are the two driving forces shaping the codon usage bias of a gene. ENC tends to show an inverse correlation with the CUB, *i.e*., higher the ENC, lower the CUB, and *vice versa*. S-genes were found to have lower ENC, which is in concordance with the other structural genes representing higher codon usage and thus regulating gene expression (Kandeel et al., 2020; Zhang et al., 2018; Gu et al., 2004). The ENC-GC3 plot suggests that natural selection and other factors influence the codon bias.

Further, the neutrality-based analysis revealed that natural selection dominated the mutational pressure in influencing the codon usage pattern among VOC S-genes. Previous studies also suggested adaptive evolution of the S-genes due to natural selection in shaping the CUBs. The positive selection was observed in the region that mediates host ACE2 binding in SARS-CoV-2 (Nyayanit et al., 2021; Zhang, Wei & He, 2006). Contrary to Nyayanit et al. (2020) who reported maximum effect of mutational pressure on the S-genes evolution, natural selection was the major determinant for the S-genes evolution, as revealed in the present study.

## Conclusion

With the emerging SARS-CoV-2 VOC, there is an urgent need to understand the impact of the S-gene mutations on various genomic features to identify its implications for vaccine targets. Due to limited studies on codon usage divergence in all the VOC, in the present study we have attempted to elucidate various causative factors shaping CUBs in VOC S-genes. This is the first report of CUBs patterns analysis in VOC S-genes to the best of our knowledge. In conclusion, the CUBs divergence of VOC S-gene is primarily driven by natural selection pressure, although other factors such as mutational pressure, compositional constraints, and other host factors cannot be overlooked. We believe that the information of S-gene CUBs patterns of VOC contributes toward deeper insights and its implications on evolution, adaptation and novel vaccine design strategies.

## Supplemental Information

10.7717/peerj.13562/supp-1 Supplemental Information 1 All the calculated indices for VOC S-gene and the host.

10.7717/peerj.13562/supp-2 Supplemental Information 2 SARS-CoV-2 VOC fasta sequences.

## Additional Information and Declarations

### Data Availability

The following information was supplied regarding data availability: We used publicly available datasets at NCBI Virus: https://www.ncbi.nlm.nih.gov/labs/virus/vssi/#/sars-cov-2.
